# Addition of *H*-phosphonates to quinine-derived carbonyl compounds. An unexpected C9 phosphonate–phosphate rearrangement and tandem intramolecular piperidine elimination

**DOI:** 10.3762/bjoc.10.85

**Published:** 2014-04-17

**Authors:** Łukasz Górecki, Artur Mucha, Paweł Kafarski

**Affiliations:** 1 Department of Bioorganic Chemistry, Faculty of Chemistry, Wrocław University of Technology, Wybrzeże Wyspiańskiego 27, 50-370 Wrocław, Poland

**Keywords:** carbonyl derivatives, dialkyl phosphite addition, organophosporus, phosphonate–phosphate rearrangement, quinine oxidation

## Abstract

The Abramov reaction, a base-catalyzed nucleophilic addition of dialkyl *H*-phosphonates (phosphites) to carbonyl compounds, was performed with oxidized quinine derivatives as the substrates. Homologous aldehydes obtained from the vinyl group reacted in a typical way which led to α-hydroxyphosphonates, first reported compounds containing a direct P–C bond between the quinine carbon skeleton and a phosphorus atom. For the C9 ketones a phosphonate–phosphate rearrangement, associated with a tandem elimination of the piperidine fragment, was evidenced.

## Introduction

Medicinal, organocatalytic and stereoselective properties of quinine make it the most prominent representative of *Cinchona* alkaloids [1], a group of natural compounds of a unique three-dimensional structure. The structure involves a particular arrangement of two rigid heterocyclic fragments: aromatic quinoline and chiral aliphatic quinuclidine, and a hydroxy function on the stereogenic carbon atom. Such an architecture combined with the presence of nucleophilic and electrophilic centers buried in a hydrophobic environment predestinates the molecule to asymmetric applications, such as: chiral catalysis, transition metal complexing, molecular recognition, chromatographic separation and analysis of enantiomers [2–5].

Synthetic modifications of the basic structure, motivated by an improved stereoselectivity potential of quinine, are an issue of ongoing trials [6–9]. Surprisingly, phosphorus compounds chemistry, particularity that avoiding an expansion of the core carbon skeleton [10–13], is poorly recognized and mainly involves esterification of different phosphorus acids with the *O*-9-hydroxy group [14–21]. These phosphorus esters were consecutively applied in organo- and metal-assisted catalysis [14,17–20] and NMR-monitored enantiodiscrimination [21]. According to our knowledge no example of formation of a direct C–P linkage between the quinine backbone and a phosphorus atom has been reported in the literature. Stimulated by this challenge we planned to envisage a nucleophilic addition of dialkyl phosphites to quinine-based carbonyl compounds and obtain 1-hydroxyalkylphosphonate derivatives (Abramov reaction, phospha-aldol reaction [22–23]). The scope, stereochemistry and side-reactions of the addition are described.

## Results and Discussion

**Scheme 1:**
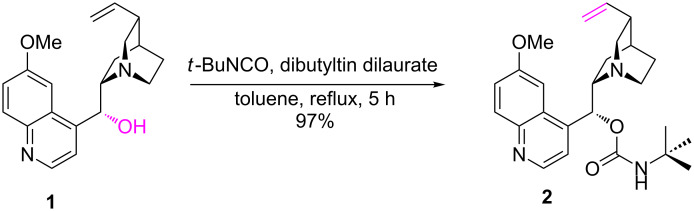
Quinine (**1**) and *O*-9-*t*-butylcarbamoylquinine (**2**) as the substrates for oxidation of the C9 hydroxy and the vinyl group, respectively.

Quinine-based carbonyl compounds were obtained by oxidation of either the secondary C9 hydroxy group to the corresponding ketone or the vinyl group to homologous aldehydes. The last-mentioned alternative demanded a protection of the OH function. This was performed via carbamoylation of quinine (**1**) with *t*-butyl isocyanate as described elsewhere (Scheme 1) [24]. A higher scale of reaction improved the yield if compared to the literature data.

### Vinyl group modifications

**Scheme 2:**
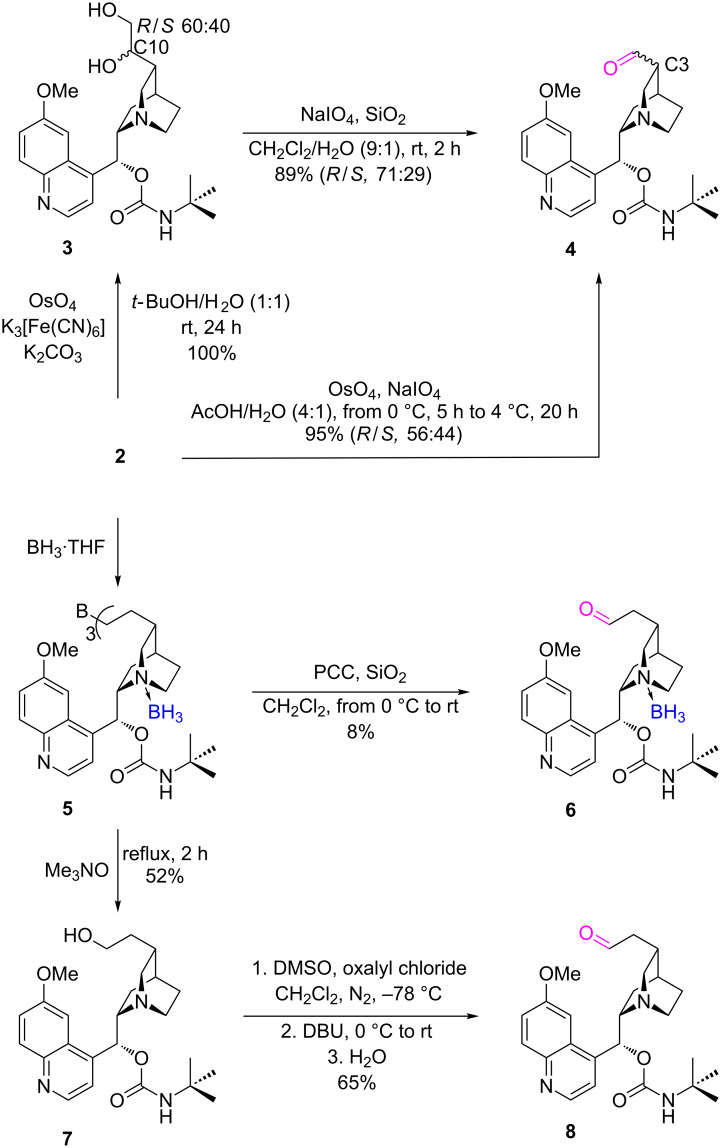
Oxidation of the vinyl group of 9-*O*-*tert*-butylcarbamoylquinine to homologous aldehydes.

Oxidation of the vinyl group of quinine can be carried out in two different manners to give homologous aldehydes. The one-carbon atom-shortened aldehyde **4** is the product of osmium tetroxide/periodate oxidation [25]. Depending on the reaction conditions a variable ratio of epimers at the neighboring C3 carbon atom was obtained (Scheme 2). A single-step oxidation process was not selective and produced equal amounts of diastereoisomers, 56:44 (C3 *R*/*S*, yield 95%), which was comparable to the literature data reported as 50:50 (C3 *R*/*S*, 95%) by Waddell [25] and 55:45 (C3 *R*/*S*, 80%) by Braje [26]. The two-step procedure initially involved the use of a co-oxidizer other than periodate, e.g., potassium hexacyanoferrate with catalytic amounts of osmium tetroxide to obtain the vicinal diol **3** [27]. This intermediate was preparatively separated as a 60:40 (*R*/*S*) mixture of epimers at C10. The diol compound was subsequently oxidized with NaIO_4_ to the aldehyde **4** with simultaneous C–C bond breakage. According to the literature an oxidative cleavage on silica in a two-phase system led predominantly to the C3 epimer of the *R* configuration 90:10 (yield 93%) in a short reaction time [27]. In our case, when the reaction time was prolonged to 2 hours, the overall yield remained at the same level while the diastereoselectivity was reduced to 71:29 (*R*/*S*).

The homologous aldehyde can be prepared by oxidation of the double bond in a hydroboration–oxidation sequence, however, the presence of the nitrogen atoms, particularly that of the tertiary amino group of quinuclidine, may be troubleshooting [28–29]. Borane complexes with heteroaromatic and aliphatic amines are considered inconveniently stable in protic solvents (water, alcohols) and dissociate only at an elevated temperature [30]. Most probably, in our case the formation of the amine–borane complex proceeded faster than the hydroboration of the vinyl group. When compound **2** was reacted with the BH_3_·THF complex and then oxidized with pyridinium chlorochromate (recommended PCC on SiO_2_ [26]) a complicated mixture of products (50% of conversion) was obtained. The mixture contained the target aldehyde **6** (minority, Scheme 2) and the corresponding alcohol (majority, ratio 1:5), both in their complexed forms (borane-tertiary amino group). Again, step-by-step approach and separation of the intermediate appeared more profitable. First, the alcohol **7** was synthesized by hydroboration of the substrate with BH_3_·THF under an inert atmosphere followed by oxidation of the intermediate borane **5** complex with trimethylamine oxide [31]. As the oxide also released the borane–quinuclidine complex at elevated temperature the free alcohol was obtained in a satisfactory yield. This alcohol was subjected to Swern oxidation, recommended for multifunctional compounds [32], to produce the target aldehyde **8** in 65% yield.

**Scheme 3:**
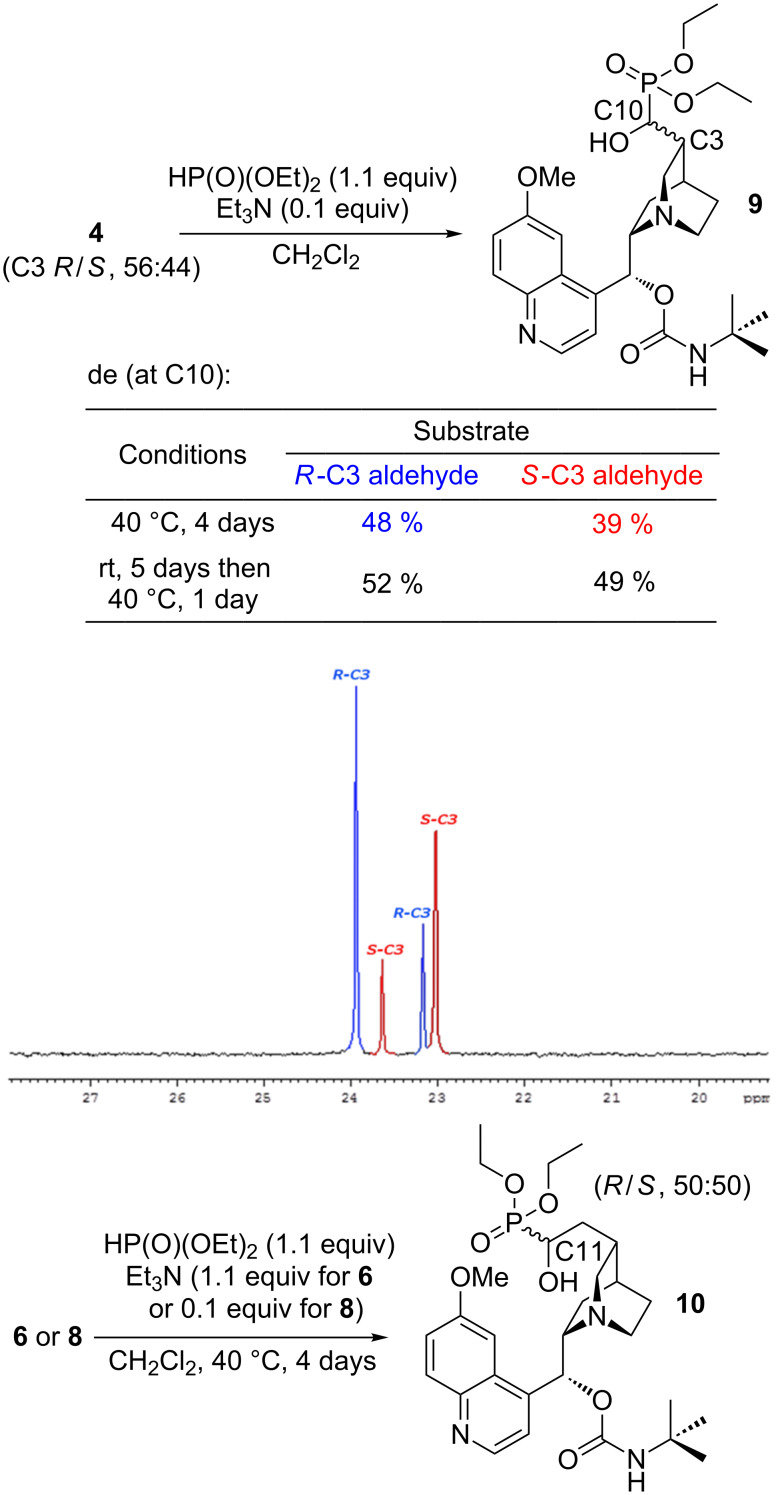
Addition of diethyl phosphite to aldehydes obtained in oxidation of the vinyl group.

The obtained aldehydes **4**, **6**, and **8** were reacted with 1.1 equiv of diethyl phosphite. The presence of the tertiary amino group of quinuclidine was expected to be a sufficient catalytic base for the addition reaction, and furthermore to induce a diastereoselectivity. However, the expected hydroxyphosphonates were not formed, neither at room temperature after 24 hours, nor upon increasing the temperature to 40 °C within additional 48 hours. Addition of 0.1 equiv of Et_3_N initiated the reaction of **4** and **8** (Scheme 3) [33], in the case of borane complex **6** a stoichiometric amount of triethylamine (1.1 equiv) was applied.

Crude reaction mixtures were analyzed by NMR. To achieve complete separation of the ^31^P NMR signals and reliable assessment of the diastereomeric composition addition of 10 equiv of acetic acid was demanded. Despite the long reaction time (up to 4 days at 40 °C) the starting aldehydes were not fully consumed. Partially stereoselective addition was observed for the shorter homolog **4**. The diastereomeric excess of the newly appearing stereogenic center at C10 of α-hydroxyphosphonate **9** slightly depended on the reaction conditions and the C3 absolute configuration of the substrate, and varied in the range of 40–50% (Scheme 3). The *R*-C3 epimer gave rise to somewhat more pronounced induction. The ^31^P NMR resonances of the predominating forms of the hydroxyphosphonate are shifted apart by approximately 1.0 ppm. We speculate that this means a diastereomeric relationship of their C3–C10 fragment (being the result of induction of the same C10 configuration, see the inserted spectrum in Scheme 3). Thus, general stereo-controlling properties of quinine predominate and do not cooperate (no match–mismatch effect visible) with the absolute configuration of the starting aldehyde epimers. The hydroxyphosphonates derived from the longer homologs were completely racemic at C11. Two diastereoisomers of the hydroxyphosphonate **10** were formed in a ratio of 1:1, irrespectively of the substrate amino group state: either free (**8**) or complexed with borane (**6**). Elevated temperature and the presence of 1.1 equiv of Et_3_N caused entire decomposition of the quinuclidine–borane complex in the case of substrate **6**. Final α-hydroxyphosphonate esters **9** and **10** were purified by column chromatography and characterized (for **9** two fractions, each containing two individuals, were refined by preparative thin-layer chromatography, for details see Supporting Information File 1).

### C9 position modification, phosphonate–phosphate rearrangement

**Scheme 4:**
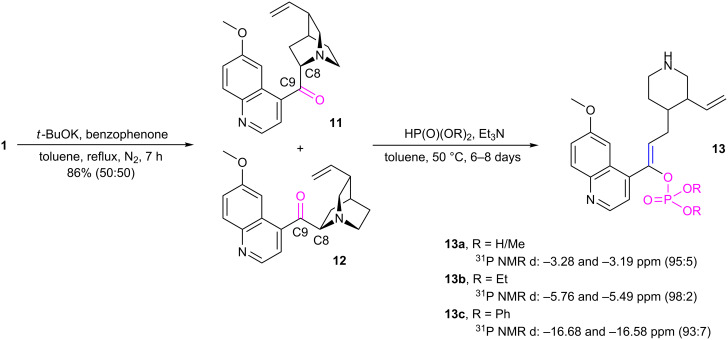
Oxidation of quinine to quininone and quinidinone and addition of phosphites to the ketones yielding the rearrangement products.

Oxidation of the C9 hydroxy group of quinine to the corresponding ketone, quininone, was performed with potassium *tert*-butoxide and benzophenone (Scheme 4) [34]. Using toluene as the solvent, instead of benzene, the reaction time was shortened to 7 hours while maintaining the same yield [35]. Epimerization, that occurred at the neighboring C8 atom, resulted in formation of two diastereomeric products: quininone **11** and quinidinone **12** in a 50:50 ratio.

The mixture of ketones **11** and **12** was treated with diethyl phosphite and heated in toluene at 50 °C for a week with addition of a catalytic amount of triethylamine (Scheme 4). The reaction mixture was purified by column chromatography. Formation of four diastereomeric compounds, derivatives of epiquinidine (8*R,*9*R*), quinidine (8*R,*9*S*), quinine (8*S,9*R) and epiquinine (8*S,*9*S*), was expected under non or partially stereoselective conditions. However, spectroscopic characterization revealed the presence of only two species (one present in an overwhelming excess) which exhibited the ^31^P NMR chemical shifts not expected for phosphonates but typical for phosphates, **13b**: −5.76 and −5.49 ppm. Apparently, they were products of the phosphonate–phosphate rearrangement of intermediate hydroxyphosphonates [36–38]. Treatment of ketones **11** and **12** with dimethyl- and diphenyl phosphite brought quite similar results. The expected product, diphenyl hydroxyphosphonate was not obtained, intead the quinotoxin enol diphenyl phosphate **13c** appeared, and it was separated chromatographically whereas methyl monodealkylated derivative **13a** precipitated directly from the reaction mixture. The selective hydrolysis of the phosphorus esters is not surprising as triethylamine and quinuclidine are bases strong enough to release the methyl ester.

**Scheme 5:**
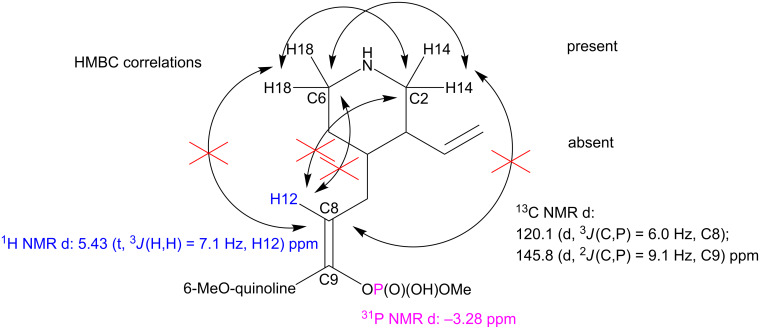
Spectroscopic features that confirmed the structure of the phosphate ester product of rearrangement and intramolecular elimination.

**Scheme 6:**
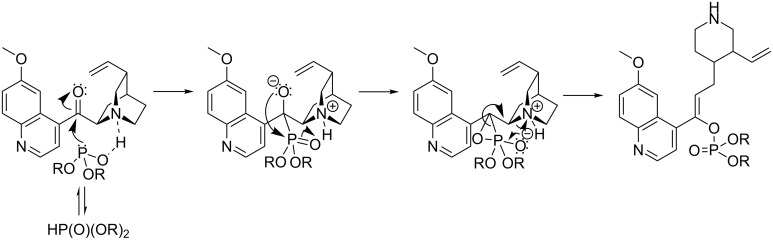
Tentative mechanism of the phosphonate–phosphate rearrangement associated with tandem quinuclidine degradation.

The additional structural modifications of the quinine skeleton of **13a** were indicated with the ^1^H,^13^C-HMBC correlation spectra. The C2–H18 and C6–H14 interactions were present, whereas correlations C2–H12, C6–H12, C8–H14 and C8–H18 were not visible (Scheme 5), what demonstrated a degradation of the bicyclic fragment of quinuclidine to a piperidine skeleton. In addition, the characteristic signal of the H11 proton was absent and the H12 resonance was shifted to the lower field (5.43 ppm), between the H20 and H21 vinyl protons. The C8 resonance signal was consequently shifted from 60 ppm to approximately 120 ppm. The aromatic system remained intact. These data suggest formation of the C8=C9 double bond in a cascade process with concomitant cleavage of the C–N bond that follows the phosphonate–phosphate rearrangement (Scheme 6). Two ^31^P NMR signals are related to the *E*/*Z* diasteroisomerism. Configuration of the predominating form can be assigned as *Z*. First, this is indicated by the nuclear Overhauser effect – irradiation of the H12 proton caused the most significant cross-relaxation changes in intensity of the H3’ and H5’ protons of the quinoline system. This proximity is achievable only in the case of location of vinyl and quinoline protons at the same side of the double bond. Theoretical prediction of the H12 NMR chemical shift provided an additional confirmation [39]. The δ calculated for the *Z* arrangement (geminal alkyl, *cis* aromatic and *trans* dialkyl phosphoryl, whose estimated influence corresponds to the acetoxy group [40]) is 5.4–5.5 ppm and well matches with the observed values (5.43–5.49). The chemical shift calculation performed for the opposite configuration remains in worse agreement (5.2–5.3 ppm).

The observed reactivity seemed to be general as formation of compound **13b** was evidenced (to a different extent) in other variants of the catalytical addition of diethyl phosphite to quininone/quinidinone, with catalytic systems such as: KF/Al_2_O_3_, NH_3_/EtOH and DBU/EtOH or toluene. Independent of the catalyst and conditions applied α-hydroxyphosphonates were not detected in the crude reaction mixture, and the rearranged compound was the only appearing product. The enol phosphates **13** were not stable and underwent slow decomposition to give four to five signals in the ^31^P NMR spectra after a month.

This is a novel contribution to the reactivity of quinine although similar eliminations of piperidine in *Cinchona* alkaloids have been reported in the literature. Accordingly, heating of quinine or derivatives in acids provided either quino-/cinchotoxine ketones or their tautomeric enol esters, depending on the substrate structure and the reaction conditions [41–43]. The corresponding compounds were also suggested as the products of a base-catalyzed Hofmann elimination of quaternary quinuclidinium salts studied as chiral catalysts [44–45]. These unwanted rearrangement negatively influenced the stereoselective properties of the alkaloids [44–45]. An elimination associated with the phosphonate–phosphate rearrangement was also reported for other 1-hydroxyphosphonate systems [46–48].

## Conclusion

An intriguing chemical behavior of C-9 quinine-derived ketones was demonstrated in the Abramov (phospha-aldol) reaction. These carbonyl compounds reacted with dialkyl and diphenyl phosphites producing quinotoxin enol phosphates that resulted from a tandem phosphonate–phosphate rearrangement and an intramolecular piperidine elimination. It can be hypothesized that the driving force of the structural changes is the proximity of the tertiary amine nucleophilic center. Based on this supposition, a mechanism of the rearrangement was suggested. The homologous C10 and C11 aldehydes obtained by oxidation of the vinyl group reacted in a typical manner to yield α-hydroxyphosphonates, the first described quinine-derived C–P compounds.

## Supporting Information

File 1 Experimental and analytical data.

## Dedication

The contribution is dedicated to Prof. Roman Tyka on his 90^th^ anniversary.
