# Supplementary material for: Addition of H-phosphonates to quinine-derived carbonyl compounds. An unexpected C9 phosphonate–phosphate rearrangement and tandem intramolecular piperidine elimination
Source: Beilstein J Org Chem. 2014 Apr 17;10:883–9. doi: 10.3762/bjoc.10.85 (PMC3999748; doi:10.3762/bjoc.10.85)
Supplement: File 1 — Experimental and analytical data. [file Beilstein_J_Org_Chem-10-883-s001.pdf]

Supporting Information

for

Addition of *H*-phosphonates to quinine-derived carbonyl compounds. An unexpected C9 phosphonate–phosphate rearrangement and tandem intramolecular piperidine elimination

Łukasz Górecki, Artur Mucha and Paweł Kafarski*

Address: Department of Bioorganic Chemistry, Faculty of Chemistry, Wrocław University of Technology, Wybrzeże Wyspiańskiego 27, 50-370 Wrocław, Poland

Email: Paweł Kafarski* - pawel.kafarski@pwr.wroc.pl

*Corresponding author

The contribution is dedicated to Prof. Roman Tyka on his 90th anniversary.

Experimental details and spectroscopic data

Contents:

General information **S2**

Synthetic procedures and characterization data **S2**

References **S14**

General information

^{1}H NMR, ^{13}C NMR, ^{31}P NMR and 2D NMR spectra (NOESY, COSY, HSQC, HMBC) were recorded on a Bruker Avance 600 MHz spectrometer (TMS as the internal standard). Chemical shifts (δ) are reported in ppm, and coupling constants (*J*) are in Hertz (Hz). The following abbreviations were used to explain the multiplicities: s = singlet, d = doublet, t = triplet, q = quartet, m = multiplet, br = broad. HRMS data were obtained using ESI ionization. Melting points were obtained on a Boëtius instrument. Substrates were purchased from commercial sources (Sigma-Aldrich, Fluka, POCh) and used as received. Column chromatography was carried out on silica gel (Fluka 60 Å, 70–230 mesh).

Synthetic procedures and characterization data

9-*O*-*tert*-Butylcarbamoylquinine (2) was prepared as described in the literature [1], starting from 0.227 mol (73.65 g) of quinine, 0.252 mol (24.98 g) of *tert*-butyl isocyanate refluxed in 450 mL of toluene with 2 mL dibutyltin dilaurate. The product was crystallized from cyclohexane (93.09 g, yield 97%, m.p.: 120.0-121.0 °C).

HRMS (TOF MS ESI): *m/z* calcd for $C_{25}H_{34}N_3O_3^+$: 424.2600 [*M*]$^+$; found: 424.2614.

9-*O*-*tert*-Butylcarbamoyloxy-22,23-dihydro-22,23-dihydroxy-6’-methoxycinchonane (3) was prepared similarly as described in the literature for acetylated quinidine [2]. 0.5 mmol (0.13 g) of OsO_4 was added to a solution of 50 mmol (21.16 g) of **2,** 140 mmol (19.35 g) of K_2CO_3 and 140 mmol (46.10 g) of $K_3[Fe(CN)_6]$ in 500 mL of *t*-BuOH/H_2O (v/v = 1:1) under argon atmosphere. The mixture was stirred for 4 h at rt, then 500 mL of $CHCl_3$ was added. The organic layer was washed with a saturated aqueous solution of $NaHSO_3$/NaCl (1:1). $NaHCO_3$ was added to the aqueous layer and it was reextracted with $CHCl_3$. The combined

organic layers were dried over Na_2SO_4 and used without further purification in the next step (22.86 g, yield 100%, diastereomeric mixture, 60:40).

HRMS (TOF MS ESI): *m/z* calcd for $C_{25}H_{36}N_3O_5^+$: 458.2655 $[M]^+$; found: 458.2633.

9-*O*-*tert*-Butylcarbamoyloxy-6'-methoxyrubane-3-carbaldehyde (4) was prepared similarly as described in the literature for acetylated quinidine [2]. A solution of 49 mmol (22.41 g) of diastereomeric diols **3** in 400 mL of CH_2Cl_2 was added to a vigorously stirred suspension of silica gel (112 g), 63.7 mmol (13.62 g) of $NaIO_4$ in 1 L of CH_2Cl_2 and 112 mL of H_2O. The reaction mixture was stirred for 2 h at rt, then the silica gel was filtered off. The phases were separated, the organic layer was dried over $MgSO_4$ and concentrated under reduced pressure. Purification by column chromatography (acetone/AcOEt, v/v = 4:1) gave aldehyde (18.54 g, yield 89%) as a white solid.

HRMS (TOF MS ESI): *m/z* calcd for $C_{24}H_{32}N_3O_4^+$: 426.2393 $[M]^+$; found: 426.2398.

9-*O*-*tert*-Butylcarbamoyloxy-22,23-dihydro-23-hydroxyquinine (7) was prepared as described in the literature for 9-O-*tert*-butyldimethylsilyl derivative [3]. 0.1 mol (42.32 g) of **2** and 0.5 mol (500 mL) $BH_3 \cdot THF$ (1 M solution in THF) were dissolved in 350 mL of diglyme under argon atmosphere at −20 °C and left to reach room temperature. Subsequently, THF was evaporated at 50 °C and 1.5 mol (166.71 g) of trimethylamine *N*-oxide dihydrate was added to the residue. The reaction mixture was refluxed for 2 h and cooled to rt. 350 mL of AcOEt and 200 mL of water were added and the phases were separated. The water phase was reextracted with 50 mL AcOEt. The combined organic layers were dried over $MgSO_4$. After solvent evaporation the residue was purified by column chromatography (AcOEt/MeOH, from 100:0 to 50:50). The product was obtained as a white solid (23.01 g, yield 52%).

HRMS (TOF MS ESI): *m/z* calcd for $C_{25}H_{36}N_3O_4^+$: 442.2706 $[M]^+$; found: 442.2731.

9-*O-tert*-Butylcarbamoyloxy-3-formylmethyl-6'-methoxyrubane (8) was obtained in a typical procedure of Swern oxidation [4]. 12 mmol (0.94 g) of DMSO in 0.5 mL of dry CH_2Cl_2 was added to 6 mmol (0.76 g) of oxalyl chloride in 75 mL of dry CH_2Cl_2 at -80 °C. The resulting solution was stirred for 15 min, and then a solution of **7** (4 mmol, 1.76 g) in 50 mL of dry CH_2Cl_2 was added. After another 15 min 20 mmol (3.04 g) of DBU was added. The solution was left to reach room temperature, then stirred for 20 min, cooled back to -30 °C, and quenched by addition of 48 mL of water. The phases were separated and the water phase was reextracted twice with 75 mL of CH_2Cl_2. The combined organic layers were washed twice with 25 mL of 5% $NaHCO_3$/brine (v/v = 1:1) and dried over $MgSO_4$. The product was purified by column chromatography (AcOEt/MeOH, from 90:10 to 80:20) and obtained as a yellow solid (1.15 g, yield 65%).

HRMS (TOF MS ESI): *m/z* calcd for $C_{25}H_{34}N_3O_4^+$: 440.2549 [*M*]$^+$; found: 4402.2554.

General procedure for hydroxyphosphonate formation

A modification of procedure described by Kozłowski et al. was used [5]. 0.02 mmol (3 μL) of triethylamine was added to a solution of 0.2 mmol of aldehyde (**4** or **8**) and 0.22 mmol of diethyl phosphite in 1 mL of CH_2Cl_2 The solution was stirred at room temperature or at 40 °C (for the details see Scheme 3 and discussion in the main article) and then concentrated under reduced pressure. The residue was purified by column chromatography (acetone/MeOH, from 100:0 to 70:30) to yield pure phosphonates (**9** or **10**).

Diethyl (9-*O*-*tert*-butylcarbamoyloxy-6’-methoxy-3-rubane)hydroxymethylphosphonate (9)

Following the column chromatogrophay, an additional separation of diastereoisomers by preparative thin-layer chromatography (ethanol as the eluent) was performed and yielded enriched fractions. Two fractions (each containing two diastereoisomers) were selected for characterization.

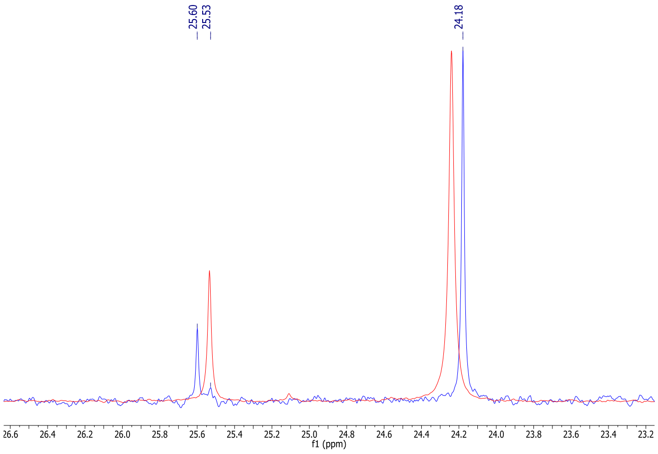

Blue fraction

HRMS (TOF MS ESI): *m/z* calcd for $C_{28}H_{43}N_3O_7P^+$: 564.2839 [*M*]$^+$; found: 564.2875.

^{31}**P NMR** (243 MHz, $CDCl_3$, 25 °C): δ = 25.60 (16%) and 24.18 ppm (84%);

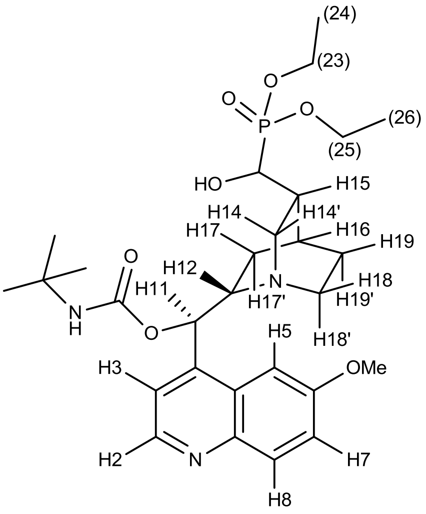

^{1}H NMR (600 MHz, $CDCl_3$, 25 °C): δ = 8.75 (d, 0.16H, H2), 8.74 (d, ^3J(H,H) = 4.5 Hz, 0.84H, H2), 8.01 (d, ^3J(H,H) = 9.2 Hz, 1H, H8), 7.47 (br s, 1H, H5), 7.37 (dd, ^3J(H,H) = 2.6 Hz, ^3J(H,H) = 9.2 Hz, 1H, H7), 7.37 (br, 1H, H3), 6.49 (br d, ^3J(H,H) = 6.5 Hz, 1H, H11), 4.77 (br s, 1H, NH_{ureth}), 4.16 (m, 4H, H23, H25), 3.96 (s, 3H, OCH_3), 3.94 (br m, 1H, CH_α), 3.34 (br m, 1H, H12), 3.12 (br m, 1H, H18'), 3.08 (br dd, H14), 2.77 (dd, ^3J(H,H) = 7.5 Hz, ^3J(H,H) = 13.8 Hz, 1H, H14'), 2.71 (br m, 1H, H18), 2.09 (br m, 1H, H16), 2.03 (br m, 1H, H15), 1.73 (br m, 3H, H17, H17', H19'), 1.49 (br m, 1H, H19), 1.33 (m, 6H, H24, H26), 1.30 ppm (br s, 9H, *t*-Bu);

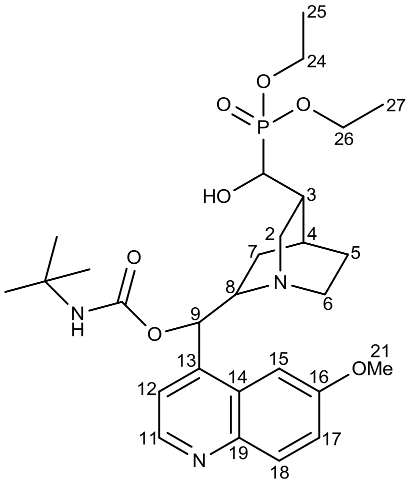

^{13}C NMR (151 MHz, $CDCl_3$, 25 °C): δ = 157.92 and 157.90 (C16), 153.85 (br, $C{=}O_{ureth}$), 147.49 (C11), 144.75 (C19), 144.47 and 144.35 (C13), 131.65 (C18), 127.38 (C14), 121.85 and 121.81 (C17), 118.68 and 118.59 (C12), 101.63 and 101.55 (C15), 72.48 (br, C9), 70.29 (d, ^1J(C,P) = 158.0 Hz, C_α), 68.64 (d, ^1J(C,P) = 158.7 Hz, C_α), 62.85* (d, ^2J(C,P) = 7.3 Hz, C24), 62.67* (d, ^2J(C,P) = 6.2 Hz, C26), 62.63 (d, ^2J(C,P) = 6.4 Hz, C24), 62.58 (d, ^2J(C,P) = 6.9 Hz, C26), 58.75 and 58.58 (C8), 55.66 (C21), 53.62 (C2), 43.03 (C6), 37.09 and 36.71 (C3), 28.88 (3 × C-*t*-Bu), 25.16 and 25.13 (C4), 22.26 and 22.17 (C5), 22.09 (C7), 16.55 ppm (br, C25, C27).

* signals of the minor isomer

Red fraction

^{31}P NMR (243 MHz, $CDCl_3$, 25 °C): δ = 25.53 (20%) and 24.24 ppm (80%);

^{1}H NMR (400 MHz, $CDCl_3$, 25 °C): δ = 8.72 (d, 0.2H, ^3J(H,H) = 4.7 Hz, H2), 8.70 (d, ^3J(H,H) = 4.7 Hz, 0.8H, H2), 8.01 (d, ^3J(H,H) = 9.2 Hz, 0.2H, H8), 8.00 (d, ^3J(H,H) = 9.2 Hz, 0.8H, H8), 7.48 (br s, 1H, H5), 7.35 (dd, ^3J(H,H) = 2.6 Hz, ^3J(H,H) = 9.2 Hz, 1H, H7), 7.35 (br, 1H, H3), 6.44 (br d, ^3J(H,H) = 6.7 Hz, 1H, H11), 4.80 (br s, 1H, NH_{ureth}), 4.15 (m, ^3J(H,H) = 7.3 Hz, 4H, H23, H25), 3.95 (s, 3H, OCH_3), 3.87 (br dd, ^3J(H,H) = 3.2 Hz, ^3J(H,H) = 8.1 Hz, 0.8H, CH_α), 3.87 (br dd, 0.2H, CH_α), 3.40 (br m, 0.8H, H12), 3.29 (br m, 0.2H, H12), 3.12 (br m, 1H, H18’), 2.99 (br m, 1H, H14’), 2.65 (br m, 1H, H14), 2.63 (br m, 1H, H18), 2.08 (br m, 1H, H16), 2.00 (br m, 1H, H15), 1.87 (br m, 1H, H17’), 1.71 (br m, 1H, H19’), 1.59 (br m, 1H, H17), 1.52 (br m, 1H, H19), 1.32 (m, 6H, H24, H26), 1.28 ppm (br s, 9H, *t*-Bu);

^{13}C NMR (151 MHz, $CDCl_3$, 25 °C): δ = 157.93* and 157.90 (C16), 153.83 (br, $C{=}O_{ureth}$), 147.38 (C11), 144.62 (br, C19), 144.30 and 144.18* (C13), 131.56* and 131.50 (C18), 127.41 (C14), 121.90 (C17), 118.66 (C12), 101.46 and 101.42* (C15), 72.36 (br, C9), 70.52 (d, ^1J(C,P) = 158.0 Hz, C_α), 69.86* (d, ^1J(C,P) = 156.0 Hz, C_α), 62.80* (d, ^2J(C,P) = 7.0 Hz, C24), 62.66* (d, ^2J(C,P) = 7.0 Hz, C26), 62.63 (d, ^2J(C,P) = 7.4 Hz, C24), 62.54 (d, ^2J(C,P) = 7.1 Hz, C26), 59.09* and 58.84 (C8), 55.67 (C21), 53.75 and 53.60 (C2), 42.41 (C6), 37.36 and 36.99 (C3), 28.83 (3 × C-*t*-Bu), 28.44 (C5), 25.23 and 25.18 (C4), 24.44 (C7), 16.53 (d, ^3J(C,P) = 1.1 Hz, C25), 16.47 ppm (d, ^3J(C,P) = 1.2 Hz, C27).

* signals of the minor isomer

Diethyl 2-[(3*R*,8*S*,9*R*)-9-*O*-*tert*-butylcarbamoyloxy-6’-methoxy-3-rubane]-1-hydroxyethylphosphonate (10, diastereomeric mixture at C_α (*R*/*S*) = 50:50**).**

HRMS (TOF MS ESI): *m*/*z* calcd for $C_{29}H_{45}N_3O_7P^+$: 578.2995 [*M*]$^+$; found: 578.2993.

^{31}P NMR (121 MHz, $CDCl_3$, 25 °C): δ = 24.24 (50%) and 24.17 ppm (50%);

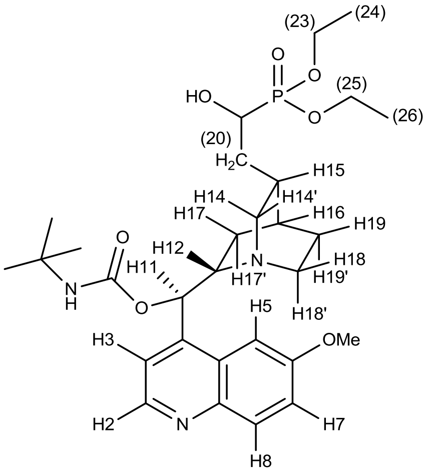

^{1}H NMR (600 MHz, $CDCl_3$, 25 °C): δ = 8.70 (br t, ^{3}J(H,H) = 3.6 Hz, 1H, H2), 7.99 (d, ^{3}J(H,H) = 9.2 Hz, 0.5H, H8), 7.98 (d, ^{3}J(H,H) = 9.2 Hz, 0.5H, H8), 7.49 (br s, 1H, H5), 7.35 (br, 1H, H3), 7.33 (br, 1H, H7), 6.52 (br, 1H, H11), 4.97 (s, 0.5H, NH_{ureth}), 4.95 (s, 0.5H, NH_{ureth}), 4.10 (m, 4H, H23, H25), 3.96 (s, 3H, OCH_3), 3.85 (br m, 0.5H, CH_{α}), 3.81 (br m, 0.5H, CH_{α}), 3.28 (br m, 1H, H12), 3.13 (br m, 2H, H18', H14), 2.67 (br m, 1H, H18), 2.48 (d, ^{3}J(H,H) = 11.9 Hz, 0.5H, H14'), 2.39 (d, ^{3}J(H,H) = 11.9 Hz, 0.5H, H14'), 1.99 (br m, 1H, H16), 1.83 (s, 0.5H, H15), 1.81 (s, 0.5H, H15), 1.74 (br m, 2H, H20), 1.67 (br m, 3H, H17, H17', H19'), 1.52 (br m, 1H, H19), 1.27 (d, 9H, *t*-Bu), 1.25 ppm (m, 6H, H24, H26);

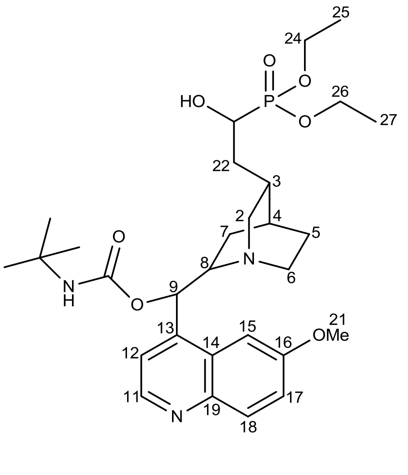

^{13}C NMR (151 MHz, $CDCl_3$, 25 °C): δ = 158.19 (C16), 153.46 (br, $C{=}O_{ureth}$), 147.18 (C11), 144.53 (C19), 144.09 (C13), 131.41 (C18), 127.13 (C14), 122.14 (C17), 118.43 (C12), 101.45 (C15), 71.97 (br, C9), 66.12 (d, ^{1}J(C,P) = 161.6 Hz, C_{α}), 65.32 (d, ^{1}J(C,P) = 162.6 Hz, C_{α}),

62.63 (d, ^2J(C,P) = 6.7 Hz, C24, C26), 58.87 (C8), 58.76 (C8), 55.96 (C21), 50.73 (C2), 42.44 (C6), 42.37 (C6), 36.1 (d, ^2J(C,P) = 95.6 Hz, C22), 31.55 (d, ^3J(C,P) = 13.8 Hz, C3), 31.03 (d, ^3J(C,P) = 13.4 Hz, C3), 28.85 (3 × C-*t*-Bu), 27.86 (C4), 27.60 (C4), 26.72 (C5), 24.77 (C5), 22.95 (C7), 22.65 (C7), 16.48 ppm (d, ^3J(C,P) = 4.1 Hz, C25, C27).

Quininone (11) and quinidinone (12) were prepared as described in the literature [6]. 150 mmol (27.33 g) of benzophenone and 75 mmol (8.42 g) of potassium *tert*-butoxide were added to the solution of 30 mmol (9.73 g) of quinine in 100 mL of toluene under nitrogen atmosphere. The mixture was refluxed for 7 h and stirred at room temperature for additional 12 h, then washed with 50 mL of 5% $NaHCO_3$/brine (v/v = 1:1) and dried over $MgSO_4$. The drying agent was filtered off and the solution was left for crystallization during slow evaporation of the solvent. The product was obtained as a light-yellow solid (8.33 g, yield 86%, m.p.: 106.5-108.0 °C), 50:50 mixture of epimers at C8.
HRMS (TOF MS ESI): *m/z* calcd for $C_{20}H_{23}N_2O_2^+$: 323.1760 [*M*]$^+$; found: 323.1757.

Quinotoxin 9-*O*-hydroxymethoxyphosphorylenol, 1-*O*-hydroxymethoxyphosphoryl-1-(6-methoxy-4-quinoline)-3-(3-vinyl-4-piperidinyl)-1-propen-1-ol (13a). Dimethyl phosphite (0.15 mL, 1.65 mmol) and triethylamine (0.10 mL, 0.75 mmol) were added to the mixture of quininone and quinidinone (**11** and **12**) (0.48 g, 1.5 mmol) disolved in 2 mL of toluene. The mixture was left for 4 days at 50 °C and then the volatile components were evaporated in vacuo. 4 mL of $CHCl_3$ was added to the residue and a precipitation occurred. The white crystalline product (0.13 g, yield 21%, m.p.: 232.0-232.5 °C) was collected by filtation.
HRMS (TOF MS ESI): *m/z* calcd for $C_{21}H_{28}N_2O_5P^+$: 419.1736 [*M*]$^+$; found: 419.1739.
^{31}P NMR (243 MHz, CD_3OD, 25 °C): δ = -3.28 ppm;

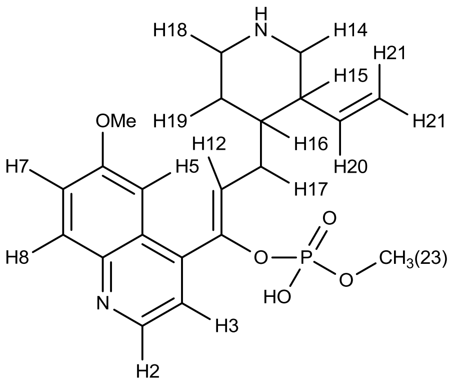

^{1}H NMR (600 MHz, CD_3OD, 25 °C): δ = 8.64 (d, ^3J(H,H) = 4.4 Hz, 1H, H2), 7.92 (d, ^3J(H,H) = 9.2 Hz, 1H, H8), 7.70 (d, ^3J(H,H) = 2.0 Hz, 1H, H5), 7.53 (d, ^3J(H,H) = 4.4 Hz, 1H, H3), 7.40 (dd, ^4J(H,H) = 2.2 Hz, ^3J(H,H) = 9.1 Hz, 1H, H7), 6.23 (m, 1H, H20), 5.43 (t, ^3J(H,H) = 7.1 Hz, 1H, H12), 5.18 (m, 2H, H21), 3.97 (s, 3H, OCH_3), 3.23 (d, ^3J(H,P) = 11.1 Hz, 3H, H23), 3.10 (br m, 1H, H18'), 2.96 (dd, ^3J(H,H) = 3.5 Hz, ^3J(H,H) = 12.6 Hz, 1H, H14), 2.86 (dd, ^3J(H,H) = 2.9 Hz, ^3J(H,H) = 12.7 Hz, 1H, H14), 2.67 (m, 1H, H18), 2.56 (m, 1H, H17), 2.38 (m, 2H, H15, H17), 1.94 (br m, 1H, H16), 1.63 ppm (m, 2H, H19);

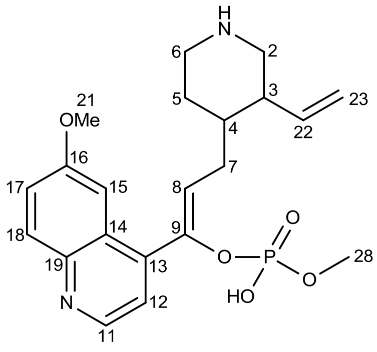

^{13}C NMR (151 MHz, CD_3OD, 25 °C): δ = 157.9 (C16), 146.7 (C11), 145.8 (d, ^2J(C,P) = 9.1 Hz, C9), 144.3 (C13), 143.6 (C19), 137.4 (C22), 129.2 (C18), 127.5 (C14), 122.1 (C17), 121.0 (C12), 120.1 (d, ^3J(C,P) = 6.0 Hz, C8), 115.6 (C23), 104.1 (C15), 54.7 (C21), 51.8 (d, ^2J(C,P) = 6.0 Hz, C28), 50.2 (C2), 45.0 (C6), 43.3 (C3), 38.7 (C4), 29.1 (C7), 28.4 ppm (C5).

Quinotoxin 9-*O*-diethoxyphosphorylenol, 1-*O*-diethoxyphosphoryl-1-(6-methoxy-4-quinoline)-3-(3-vinyl-4-piperidinyl)-1-propen-1-ol (13b). Diethyl phosphite (0.85 mL, 6.6

mmol) and triethylamine (0.42 mL, 3 mmol) were added to the mixture of quininone and quinidinone (**11** and **12**, 1.93 g, 6 mmol) disolved in 10 mL of toluene. The mixture was left for 6 days at 50 °C and then the volatile components were evaporated in vacuo. The residue was purified by column chromatography on silica gel with a mixture of solvents AcOEt/MeOH/Et_3N (81.0:18.0:1.0) as the eluent. The product was obtained after concentration of appropriate fractions as a pale-yellow oil (0.58 g, yield 21%).

HRMS (TOF MS ESI): *m/z* calcd for $C_{24}H_{34}N_2O_5P^+$: 461.2205 $[M]^+$; found: 461.2220.

^{31}P NMR (243 MHz, $CDCl_3$, 25 °C): δ = -5.76 ppm;

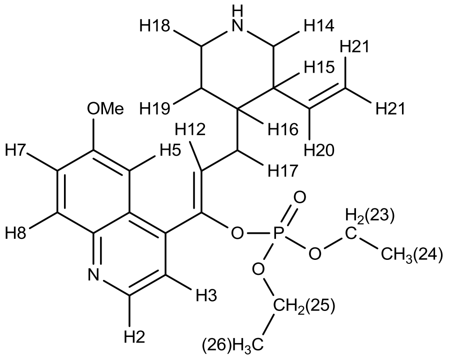

^{1}H NMR (600 MHz, CD_3OD, 25 °C): δ = 8.75 (d, ^3J(H,H) = 4.4 Hz, 1H, H2), 8.02 (d, ^3J(H,H) = 9.2 Hz, 1H, H8), 7.41 (d, ^3J(H,H) = 2.7 Hz, 1H, H5), 7.39 (dd, ^4J(H,H) = 2.7 Hz, ^3J(H,H) = 9.1 Hz, 1H, H7), 7.37 (d, ^3J(H,H) = 4.4 Hz, 1H, H3), 6.15 (br m, 1H, H20), 5.49 (t, ^3J(H,H) = 7.4 Hz, 1H, H12), 5.16 (m, 2H, H21), 3.94 (m, 2H, H23), 3.93 (s, 3H, OCH_3), 3.90 (m, 2H, H25), 2.98 (br m, 1H, H18’), 2.83 (br m, 1H, H14), 2.62 (br m, 1H, H14), 2.49 (br m, 2H, H18, H15), 2.36 (m, 2H, H17), 1.84 (br m, 1H, H16), 1.79 (m, 2H, H19), 1.08 (dt, ^3J(H,H) = 1.0 Hz, ^3J(H,H) = 7.1 Hz, 3H, H24), 1.03 ppm (dt, ^3J(H,H) = 1.0 Hz, ^3J(H,H) = 7.1 Hz, 3H, H26).

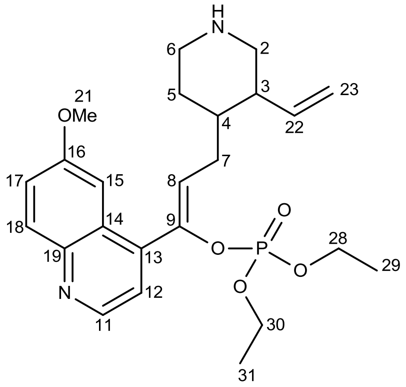

^{13}C NMR (151 MHz, $CDCl_3$, 25 °C): δ = 158.0 (C16), 147.4 (C11), 144.7 (C9), 144.4 (C13), 140.7 (C19), 137.6 (C22), 131.3 (C18), 126.9 (C14), 122.2 (C17), 121.5 (d, ^3J(C,P) = 6.6 Hz, C8), 121.3 (C12), 116.5 (C23), 103.4 (C15), 64.3 (m, C28, C30), 55.5 (C21), 52.6 (C2), 42.4 (C6), 39.7 (C3), 37.8 (C4), 27.8 (C7), 27.5 (C5),15.8 ppm (m, C29, C31).

Quinotoxin 9-*O*-diphenoxyphosphorylenol, 1-*O*-diphenoxyphosphoryl-1-(6-methoxy-4-quinoline)-3-(3-vinyl-4-piperidinyl)-1-propen-1-ol (13b). Diphenyl phosphite (1.00 mL, 4.4 mmol) and triethylamine (0.28 mL, 2 mmol) were added to the mixture of quininone and quinidinone (**11** and **12**) (1.29 g, 4 mmol) disolved in 5 mL of toluene. The mixture was left for 6 days at 50 °C and then the volatile components were evaporated *in vacuo*. The residue was purified by column chromatography on silica gel with a mixture of solvents $CHCl_3$/MeOH/NH_4OH (a gradient from 98.5:1.0:0.5 to 87.0:12.5:0.5) as the eluent. The product was obtained after concentration of appropriate fractions and precipitated from the residue with Et_2O. It was filtered as a white solid (0.40 g, yield 18%, m.p.: 205.5-206.0 °C).

HRMS (TOF MS ESI): *m/z* calcd for $C_{32}H_{34}N_2O_5P^+$: 557.2205 [*M*]$^+$; found: 557.2184.

^{31}P NMR (243 MHz, $CDCl_3$, 25 °C): δ = -16.68 ppm;

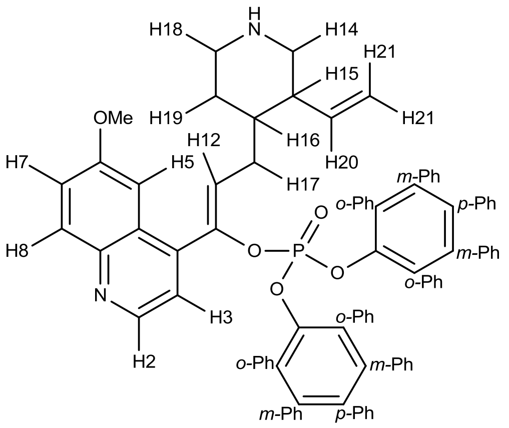

^{1}H NMR (600 MHz, $CDCl_3$, 25 °C): δ = 8.70 (d, ^3J(H,H) = 4.4 Hz, 1H, H2), 8.00 (d, ^3J(H,H) = 9.2 Hz, 1H, H8), 7.37 (dd, ^4J(H,H) = 2.2 Hz, ^3J(H,H) = 9.1 Hz, 1H, H7), 7.33 (d, ^3J(H,H) = 4.4 Hz, 1H, H3), 7.27 (d, ^3J(H,H) = 2.0 Hz, 1H, H5), 7.22 (m, 4H, *m*-Ph), 7.14 (m, 2H, *p*-Ph), 6.96 (d, ^3J(H,H) = 8.4 Hz, 2H, *o*-Ph), 6.91 (d, ^3J(H,H) = 8.4 Hz, 2H, *o*-Ph), 6.09 (m, 1H, H20), 5.48 (t, ^3J(H,H) = 7.2 Hz, 1H, H12), 5.23 (m, 2H, H21), 3.85 (s, 3H, OCH_3), 3.31 (m, 1H, H18'), 3.13 (dd, ^3J(H,H) = 3.6 Hz, ^3J(H,H) = 12.6 Hz, 1H, H14), 3.07 (dd, ^3J(H,H) = 5.0 Hz, ^3J(H,H) = 13.2 Hz, 1H, H14), 3.00 (m, 1H, H18), 2.72 (br, 1H, H15), 2.29 (m, 2H, H17), 1.96 (br m, 1H, H16), 1.88 ppm (m, 2H, H19);

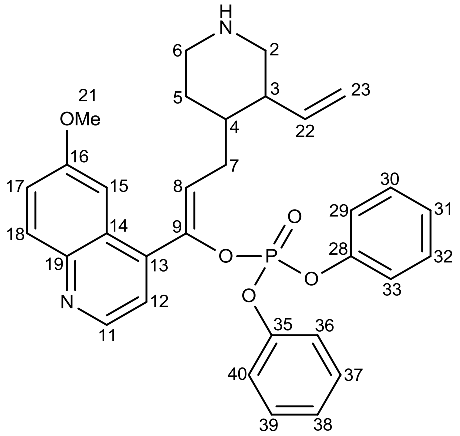

^{13}C NMR (151 MHz, $CDCl_3$, 25 °C): δ = 158.1 (C16), 150.0 (d, ^2J(C,P) = 4.5 Hz, 2C, C28, C35), 147.3 (C11), 145.1 (d, ^2J(C,P) = 9.1 Hz, C9), 144.7 (C13), 139.4 (C19), 133.9 (C22), 131.3 (C18), 129.8 (2C, C30, C32), 129.8 (2C, C37, C39), 126.8 (C14), 125.7 (C31), 125.6 (C38), 122.3 (C17), 121.6 (C12), 120.8 (d, ^3J(C,P) = 7.6 Hz, C8), 119.7 (2C, C29, C33),

119.5 (2C, C36, C40), 119.2 (C23), 103.2 (C15), 55.5 (C21), 46.4 (C2), 42.3 (C6), 40.2 (C3), 36.3 (C4), 27.0 (C7), 24.8 ppm (C5).

References

1. Scully, N. M.; O'Sullivan, G. P.; Healy, L. O.; Glennon, J. D.; Dietrich, B.; Albert, K. *J. Chromatogr. A* **2007**, *1156*, 68-74.

2. Braje, W.; Frackenpohl, J.; Langer, P.; Hoffmann, H. M. R. *Tetrahedron* **1998**, *54*, 3495-3512.

3. Rowan, S. J.; Sanders, J. K. M. *J. Org. Chem.* **1998**, *63*, 1536-1546.

4. Smith, A. B., III; Liu, H.; Hirschmann, R. *Org. Lett.* **2000**, *2*, 2037-2040.

5. Kozlowski, J. K.; Rath, N. P.; Spilling, C. D. *Tetrahedron* **1995**, *51*, 6385-6396.

6. Hutchison, D. R.; Khau, V. V.; Martinelli, M. J.; Nayyar, N. K.; Peterson, B. C.; Sullivan, K A. *Org. Synth.* **1998**, *75*, 223-234.
